# Absence of dry season *Plasmodium* parasitaemia, but high rates of reported acute respiratory infection and diarrhoea in preschool-aged children in Kaédi, southern Mauritania

**DOI:** 10.1186/1756-3305-5-193

**Published:** 2012-09-07

**Authors:** Sunkaru Touray, Hampâté Bâ, Ousmane Bâ, Mohamedou Koïta, Cheikh B Ould Ahmed Salem, Moussa Keïta, Doulo Traoré, Ibrahima Sy, Mirko S Winkler, Jürg Utzinger, Guéladio Cissé

**Affiliations:** 1 Department of Epidemiology and Public Health, Swiss Tropical and Public Health Institute, P.O. Box,, CH-4002, Basel, Switzerland; 2 University of Basel, P.O. Box,, CH-4003, Basel, Switzerland; 3 Institut National de Recherche en Santé Publique, BP 695, Nouakchott, Mauritania

**Keywords:** Malaria, Acute respiratory infection, Diarrhoea, Cross-sectional survey, Dry season, Mauritania

## Abstract

**Background:**

The epidemiology of malaria in the Senegal River Gorgol valley, southern Mauritania, requires particular attention in the face of ongoing and predicted environmental and climate changes. While “malaria cases” are reported in health facilities throughout the year, past and current climatic and ecological conditions do not favour transmission in the dry season (lack of rainfall and very high temperatures). Moreover, entomological investigations in neighbouring regions point to an absence of malaria transmission in mosquito vectors in the dry season. Because the clinical signs of malaria are non-specific and overlap with those of other diseases (e.g. acute respiratory infections and diarrhoea), new research is needed to better understand malaria transmission patterns in this region to improve adaptive, preventive and curative measures.

**Methods:**

We conducted a multipurpose cross-sectional survey in the city of Kaédi in April 2011 (dry season), assessing three major disease patterns, including malaria. *Plasmodium* spp. parasite rates were tested among children aged 6–59 months who were recruited from a random selection of households using a rapid diagnostic test and microscopic examination of Giemsa-stained thick and thin blood films. Acute respiratory infection and diarrhoea were the two other diseases investigated, administering a parental questionnaire to determine the reported prevalence among participating children.

**Findings:**

No *Plasmodium* infection was found in any of the 371 surveyed preschool-aged children using two different diagnostic methods. Acute respiratory infections and diarrhoea were reported in 43.4% and 35.0% of the participants, respectively. About two thirds of the children with acute respiratory infections and diarrhoea required medical follow-up by a health worker.

**Conclusions:**

Malaria was absent in the present dry season survey in the capital of the Gorgol valley of Mauritania, while acute respiratory infections and diarrhea were highly prevalent. Surveys should be repeated towards the end of rainy season, which will enhance our understanding of the potential changes in malaria transmission in a region known as ‘hot spot’ of predicted climate change.

## Background

Malaria continues to be a major health challenge globally with an estimated 655,000 to 1.2 million deaths in 2010 [1,2]. About 90% of the malaria-related deaths have been reported from sub-Saharan Africa [2]. In Mauritania, malaria has increased in recent years, but it is confined to the southern and south-eastern parts of the country, where about 60% of the resident population are at risk of infection with *Plasmodium falciparum*[2,3]. The nature of malaria transmission in the Senegal River Gorgol valley, southern Mauritania, is poorly understood due to the lack of epidemiological data [2-4], and also because many febrile presentations are attributed to malaria without differential diagnosis, and hence are treated presumptively [3,4]. Since “malaria cases” are reported in health facilities in the southern part of the country throughout the year, some consider the area a perennial transmission zone [3,5]. Conversely, as the area is characterized by two distinct seasons (rainy season and dry season), it is suspected that during the dry season when there is no rainfall and temperatures are very high (>45°C), malaria transmission is likely to be absent [6].

Recent entomological investigations have identified *Anopheles arabiensis* as the predominant vector species in the region, while *An*. *pharoensis* and *An. funestus* were reported in close proximity to irrigation schemes [7]. However, due to a lack of reliable health information and resources to confirm presumptive clinical cases [3,4], further investigations are needed to clarify the transmission pattern of malaria in the southern parts of Mauritania. Preceding studies revealed little or no transmission among vectors in the dry season [8] and reports on *P. falciparum* parasite rates (*Pf*PR) are scarce and conflicting [3,9]. Whether laboratory-confirmed cases in the dry season are autochthonous or imported from other regions is also currently unknown, because data are collected from health facilities serving populations from various regions in Mauritania as well as neighbouring Senegal.

By studying *Plasmodium* parasitaemia rates in the human population in the dry season, comparisons can be made with entomological studies and laboratory data that could contribute to a better understanding of malaria transmission in the Gorgol valley. Of note, most published studies in the region on malaria are dated, they were primarily based on hospital data, and only few attempts have been made to characterize malaria infection in the preschool-aged population in the dry season. To contribute to filling in these gaps, we conducted a multipurpose study, including a determination of malaria infection among a random sample of children aged 6–59 months who were recruited during a cross-sectional household survey in the dry season of 2011.

## Methods

### Ethical considerations

Approval for this study was obtained from the ethics committee in Basel, Switzerland (EKBB, reference no. 117/11) and the national ethics committee of the Ministry of Health in Mauritania (reference no. 00158). Local government and health administrative authorities were involved in the planning, approval and announcement of the objectives and procedures of the study to the local residents. Parents (or legal guardians) of participating children signed a written informed consent sheet before finger prick blood samples were taken and questionnaires administered.

### Study area and population

Our study was carried out in March 2011 in the city of Kaédi, southern Mauritania. The design was cross-sectional, and we aimed at recruiting approximately 350 children aged 6–59 months from various communes in Kaédi. We randomly selected 246 households, following a method described elsewhere [10]. In brief, we started at a central location by selecting the first household using a direction determined by spinning a pen and then selected the first household along the direction pointing outwards. The next household adjacent to the initially selected one was invited for participation and subsequent households were selected iteratively through door-to-door visits. All children in the required age range in each household were eligible for participation. The geographical coordinates of each household were recorded using a hand-held global positioning system (GPS; Garmin eTrex H Handheld GPS Navigator).

### Field and laboratory procedures

A finger prick blood sample was collected and a malaria rapid diagnostic test (RDT) was administered according to the manufacturer’s instructions (Optimal-IT® DiaMed; Basel, Switzerland). Two additional drops of blood were used for thick and thin film preparations on microscope slides. The RDT results were read out on site, while the slides were transferred to a nearby laboratory, stained with 10% Giemsa and later analyzed under a microscope by experienced technicians. Each slide was examined by two technicians, both of whom were blinded to the report of the other before final results were entered. Provisions were made for treatment of any positive cases using national guidelines with an artemisinin-based combination therapy (ACT).

### Questionnaire survey

For each child recruited during the survey, a questionnaire was administered to the mother or legal guardian of the child, enquiring about the occurrence of symptoms of acute respiratory infections and diarrhoea in the preceding 2 weeks, using definitions put forth by the World Health Organization (WHO) [11,12].

## Results and discussion

**Figure 1:**
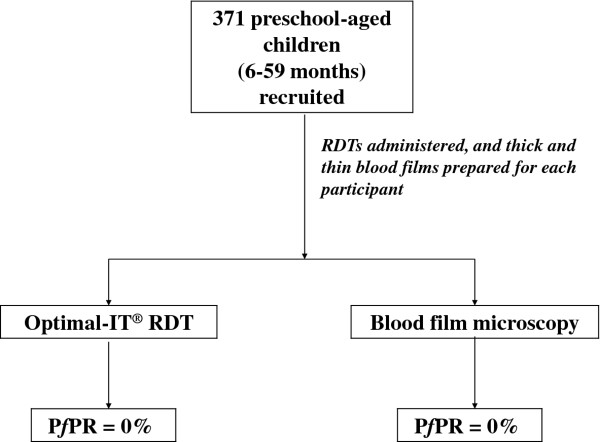
Flow chart depicting participant recruitment and malaria test administration.

**Figure 2:**
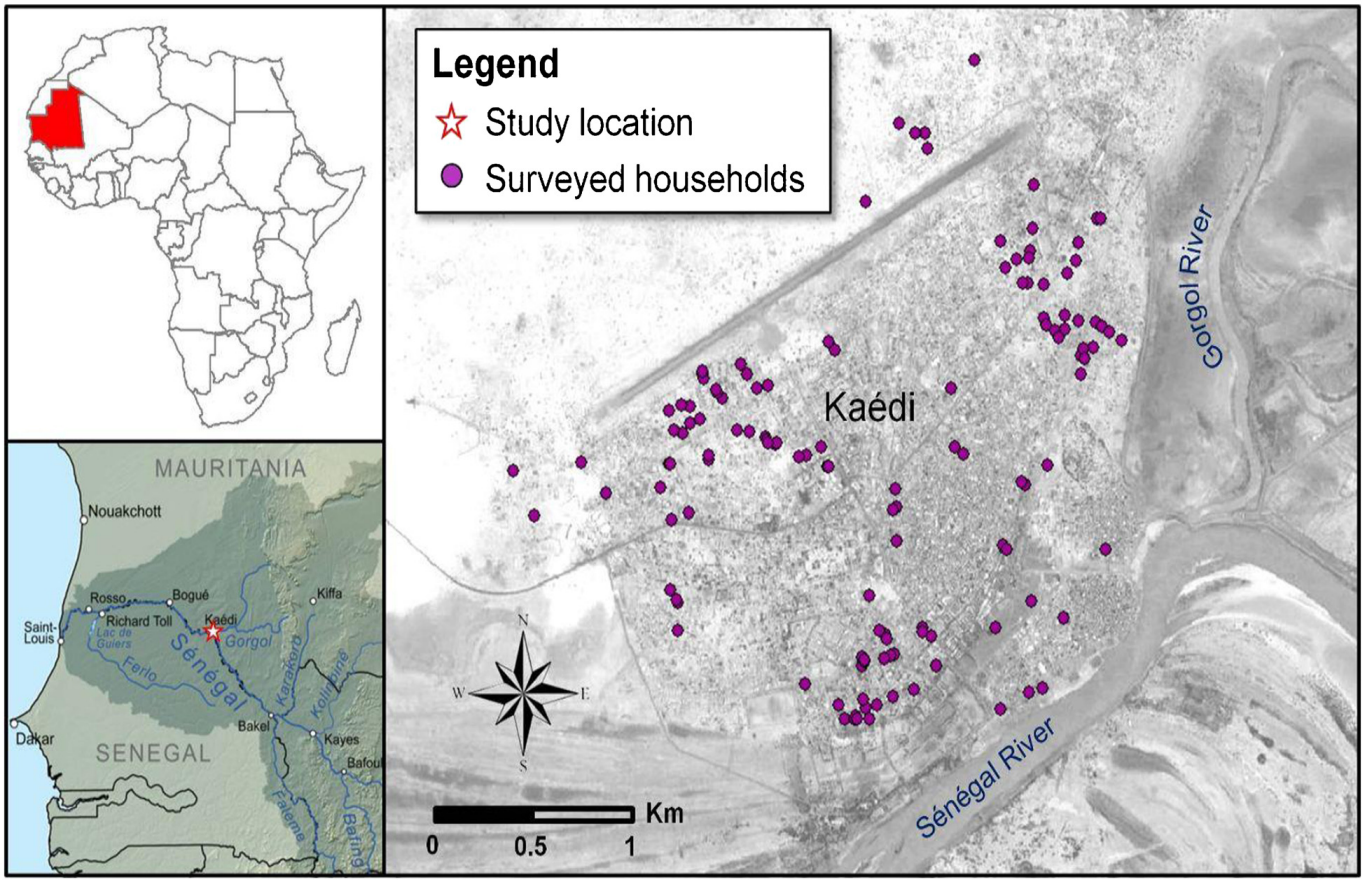
Map of study site with selected geo-referenced households.

As shown in Figure 1, 371 children were recruited in the required age range of 6–59 months from 246 randomly selected households in various communes in the city of Kaédi. Figure 2 shows the geographical location of the households from which participants were recruited. The malaria prevalence rate was 0%. Indeed, neither the RDT nor the microscopic diagnosis of Giemsa-stained blood films revealed a *P. falciparum* infection among the 371 preschool-aged children.

It is important to note that clinical signs and symptoms of malaria are non-specific and fever is a common presentation that overlaps with other clinical entities such as respiratory tract infections and gastrointestinal illnesses [13]. We additionally enquired about the occurrence of acute respiratory infection and diarrhoea in our study sample. Hence, mothers/guardians were interviewed about these diseases using a recall period of 2 weeks. We found high frequencies of acute respiratory infection and diarrhoea, 43% and 35% of the mothers/guardians interviewed reported these conditions, respectively. About two thirds of the children with acute respiratory infection and diarrhoea required further assessment by medical staff with no significant sex difference in the occurrence of these signs among participants (Table 1). 

**Table 1:** Prevalence of acute respiratory infections and diarrhoea, as determined by a questionnaire administered to mothers/guardians of children aged 6–59 months in Kaédi, southern Mauritania in March 2011, stratified by sex

**Disease**	**No. of children interviewed**	**No. of children tested positive (%)**	**No. of children needing further assessment (%)**	**Number (%) of children tested positive, stratified by sex**		**χ**^**2**^	**P-value**
				Males (n = 199)	Females (n = 172)		
Acute respiratory infections	371	161 (43.4)	105 (65.2)	84 (42.2)	77 (44.8)	0.25	0.620
Diarrhoea	371	130 (35.0)	87 (66.9)	63 (31.7)	67 (39.0)	2.16	0.142

Our observation of a nil P*f*PR concurs with recent findings obtained in entomological investigations in neighbouring regions in the dry season of 2009, as reported by Ndiath and colleagues [8]. Indeed, in the longitudinal arm of their study, the authors examined more than 2,400 *Anopheles* specimens during a 16-month period with an overall infection rate of 1.1%. No infected mosquitoes were observed between January and June 2009. However, some transmission was observed in January of 2010, albeit at low levels. Malaria transmission may occur between November and January because, in the absence of rainfall, the annual flood recession from the Gorgol River and the Senegal River during these months can provide residual breeding sites for mosquitoes. Our observations are also consistent with older studies in the Gorgol region conducted shortly after the filling of the Foum Glieta dam, as Baudon and colleagues reported the absence of malaria transmission in March during the dry season [9]. Rainfall time-series data for Kaédi show a high inter-annual variation, with unpredictable changes in the duration of the rainy season and amount of rainfall experienced. The absence of parasitaemia in the general population can be explained in part by the absence of rainfall coupled with high average temperatures in the city that can exceed 45°C. Clearly such climatic factors do not favour malaria transmission. In their study, Ndiath and colleagues [8] also noted a considerable difference in the number of mosquitoes caught in January 2009 (dry season) compared to October (rainy season) in the same year (27 *versus* 568 mosquitoes). In 2009, Dia and co-workers [7] found a low *Plasmodium* infection rate when they examined 394 *An. arabiensis* mosquitoes with only one specimen being infected with *P. falciparum*, owing to an infection rate of 0.25%*.* Furthermore, there was a considerable difference in infection rates of mosquitoes in these same months (0% compared to 1.06%), suggesting that transmission appears to occur only during the rainy season [8]. Unpredictable trends in the duration of the rainy season, and the amount of rainfall due to climate variability and change highlight the need for more appropriate public health efforts in malaria control.

Laboratory data from the Kaédi regional hospital report 4% slide positivity rate in April 2009 (unpublished observations), while Cortes and colleagues recorded a dry season prevalence of 16.6% in April-May 2003 [3]. Several factors could account for the variation between laboratory-reported estimates and findings in the population. Firstly, imported cases from neighbouring regions could account for this difference because the hospital is the only tertiary referral centre in the region, and hence it serves not only local residents, but also people from other regions in Mauritania as well as residents from neighbouring Senegal. The presence of highly focal transmission among residents who live in close proximity to irrigated rice fields or other social-ecological settings conducive for vector proliferation and transmission might also contribute to the observed variation. Even though studies have shown an association between higher anopheline densities in villages situated in flooded areas with ditch water as compared to those in sandy ground, a change in mosquito density does not automatically result in direct changes in malaria transmission [8,14]. Further in-depth entomological studies characterizing the geospatial and seasonal distribution as well as the infection rates in common vectors will undoubtedly help in better understanding the malaria transmission ‘hot spots’ in these areas.

## Conclusions

The absence of *Plasmodium* parasitaemia among preschool-aged children in our multi-purpose cross-sectional survey conducted in Kaédi, southern Mauritania points, once more, to very little or no malaria transmission in the dry season. However, the possibility of highly focal transmission zones cannot be discounted because local transmission may occur during the annual flood recession in the months immediately following the end of the rainy season (November to January). Between February and June when the swamps have dried up and ambient temperatures are very high, acute respiratory infections and diarrhoea appear to be the underlying aetiology in most febrile illnesses among preschool-aged children. The implications are that public health efforts to control malaria should be strengthened in the rainy season, and the months immediately following the cessation of the rains. Nevertheless the predicted climate change patterns in this region require careful surveillance of malaria transmission. The case management of febrile children under the age of 5 years should adopt a broader consideration of differential diagnosis, which should include acute respiratory infection and diarrhoea (among other bacterial and viral infections), as more likely aetiologies in the dry season. Importantly, malaria treatment with ACT should be strictly contingent on a positive malaria test (RDT or microscopy), which has been shown to be a safe and effective strategy to reduce unnecessary malarial treatment of febrile cases [15,16]. More research is needed to determine whether a recent travel history might explain positive laboratory-confirmed cases in health facilities in the dry season. This research will yield interesting insights about whether such cases are autochthonous or imported from other regions or from neighbouring countries, and might serve as a benchmark for investigating a potential impact of climate change.

## Competing interests

The authors declare that they have no competing interests.

## Authors’ contributions

ST, HB, MSW, JU and GC conceived the study. ST, HB, OB, MK and CBOAS conducted field and laboratory work. DT and IS co-coordinated the geographic survey, while MSW processed the geo-referenced data and produced the map. ST performed statistical analysis, interpreted the data and drafted the manuscript. HB, MSW, JU and GC revised the manuscript. All authors read and approved the final submitted version of the manuscript.
